# Artificial Neural Networks in Image Processing for Early Detection of Breast Cancer

**DOI:** 10.1155/2017/2610628

**Published:** 2017-04-03

**Authors:** M. M. Mehdy, P. Y. Ng, E. F. Shair, N. I. Md Saleh, C. Gomes

**Affiliations:** ^1^Department of Computer and Communication System Engineering, Universiti Putra Malaysia, Serdang, Selangor, Malaysia; ^2^Department of Electrical and Electronics Engineering, Universiti Putra Malaysia, Serdang, Selangor, Malaysia; ^3^Department of Chemical and Environmental Engineering, Universiti Putra Malaysia, Serdang, Selangor, Malaysia

## Abstract

Medical imaging techniques have widely been in use in the diagnosis and detection of breast cancer. The drawback of applying these techniques is the large time consumption in the manual diagnosis of each image pattern by a professional radiologist. Automated classifiers could substantially upgrade the diagnosis process, in terms of both accuracy and time requirement by distinguishing benign and malignant patterns automatically. Neural network (NN) plays an important role in this respect, especially in the application of breast cancer detection. Despite the large number of publications that describe the utilization of NN in various medical techniques, only a few reviews are available that guide the development of these algorithms to enhance the detection techniques with respect to specificity and sensitivity. The purpose of this review is to analyze the contents of recently published literature with special attention to techniques and states of the art of NN in medical imaging. We discuss the usage of NN in four different medical imaging applications to show that NN is not restricted to few areas of medicine. Types of NN used, along with the various types of feeding data, have been reviewed. We also address hybrid NN adaptation in breast cancer detection.

## 1. Introduction

Breast cancer is one of the main causes of death among women and the most frequently diagnosed non-skin cancer in women [1]. Breast cancer occurs when the cell tissues of the breast become abnormal and uncontrollably divided. These abnormal cells form large lump of tissues, which consequently becomes a tumor [2]. Such disorders could successfully be treated if they are detected early. Thus, it is of importance to have appropriate methods for screening the earliest signs of breast cancer.

Microcalcifications and masses are the earliest signs of breast cancer which can only be detected using modern techniques. Microcalcifications are clusters of calcium deposits which are very small in size and present inside the soft breast tissues [2]. Generally, detection of masses in breast tissues is more challenging compared to the detection of microcalcifications, not only due to the large variation in size and shape but also because masses often exhibit poor image contrast when using mammography [3]. The difficulty in classification of benign and malignant microcalcifications also causes a significant problem in medical image processing.

Automated classifiers may be useful for radiologists in distinguishing between benign and malignant patterns. Thus, in this paper, an artificial neural network (ANN) which can be served as an automated classifier is investigated. In medical image processing, ANNs have been applied to a variety of data-classification and pattern recognition tasks and become a promising classification tool in breast cancer [4]. ANN applications in mammography, ultrasound, and MRI and IR imaging for early detection of breast cancer are reviewed in this paper.

Image features can be distinguished in many aspects, such as texture, color, shape, and spatial relations. They can reflect the subtle variance in many degrees. Thus, different selections of image features will result in different classification decisions. These classifications can be divided into three types: first, the method based on statistics, such as Support Vector Machine; second, the method based on rule, such as decision tree and rough sets; and third, artificial neural network [5].

In the early 1980s, there was an increment in the use of neural networks in the field of image and signal processing. The main benefit was the reduction in manipulation time due to the parallel-distributed processing behavior of neural networks [6]. Then the network had been used widely in the common image processing methods such as vector quantization, eigenvector extraction, 2D pulse code modulation, or 2D filtering [7]. The artificial neural network resembles the function of the biological neuron, and it is composed of neurons with different layers and these neurons are interconnected by numeric weights; these weights can be changed due to the learning behavior of the network to approach the optimum result. Usually in image processing applications, the number of the neurons is directly related to the number of pixels in the input image [8], and the number of layers depends on the processing steps.

For cancer detection and classification, image segmentation has been widely used. Many image segmentation methods, based on histogram features, edge detection, region growing, or pixel classification, have been trained using ANNs [9].

Although the technology related to ANN in breast cancer detection has rapidly moved forward during the last few years, there is a dearth of critical literature review on the subject which is a distinct drawback for further development of the technologies. This paper is an attempt to fulfill that vacuum in the field of image processing in the early detection of breast cancer.

## 2. Applications of ANNs

### 2.1. Mammogram

**Figure 1:**
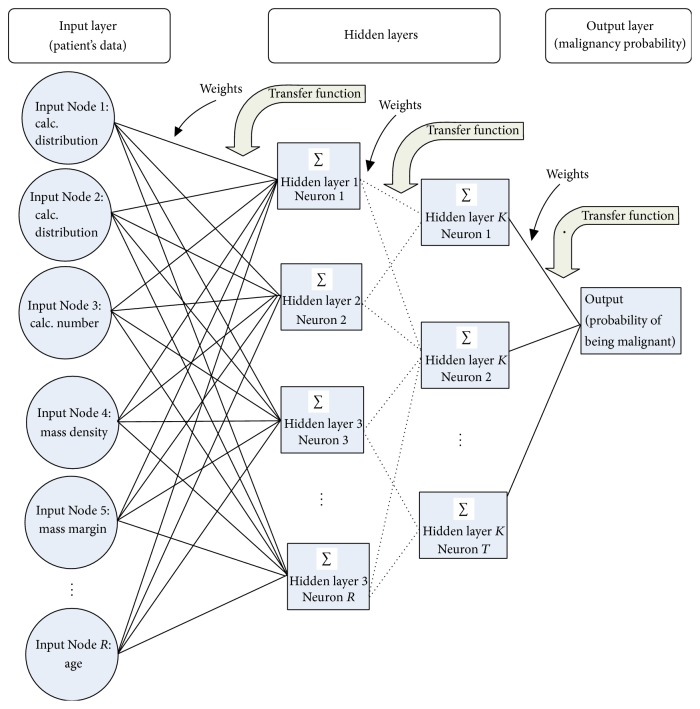
Structure of a typical ANN for classification of breast tumors in mammography [12].

Mammography is one of the most effective methods used in hospitals and clinics for early detection of breast cancer. It has been proven effective to reduce mortality as much as by 30% [3]. The main objective of screening mammography is to early detect the cancerous tumor and remove it before the establishment of metastases [3, 10, 11]. The early signs for breast cancer are masses and microcalcification but the abnormalities and normal breast tissues are often difficult to be differentiated due to their subtle appearance and ambiguous margins [3]. Only about 3% of the required information are revealed during a mammogram where a part of suspicious region is covered with vessels and normal tissues. This situation may cause the radiologists difficult to identify a cancerous tumor. Thus, computer-aided diagnosis (CAD) has been developed to overcome the limitation of mammogram and assists the radiologists to read the mammograms much better [10]. ANN model is the most commonly used in CAD for mammography interpretation and biopsy decision making. There are two ways used in ANN to assist in mammography interpretation: first, applying classifier directly to the region of interest (ROI) image data and second, understanding the situation from the features extracted from the preprocessed image signals [12].  Figure 1 shows an example of ANN structure with multi-featured input data and multi-hidden layers [12].

Microcalcification is deposition calcium in the soft breast tissues. They are quite minute in quantity and size. It is found in a cluster or pattern of circles/lines together with extra cell activity in breast region [2]. Many researchers have developed CAD system using artificial neural network to detect microcalcification. In early 90's, research done by Dhawan et al. [13] has defined image structure features using the second-order grey-level statistics. The classification was based on implementing perceptron based 3-layer neural network and the network uses backpropagation algorithm in training which has been used successfully for a number of pattern recognition and classification applications [13, 14]. The entropy feature has significant discriminating power for classification [13]. The group of researchers further extended their research in investigating the potential of using second-order histogram textural features for their correlation with malignancy. Several architectures of neural networks were proposed to analyze the features extracted from segmented calcifications and it shows that the neural network gives good results for the classification of hard-to-diagnoses cases of mammographic microcalcification into benign and malignant categories using the selected set of features [15].

Image segmentation is a technique used in image processing. Basically, segmentation is performed on the raw image to detect small, local, and bright spots. Research done by Kevin et al. [16] has drawn significant attention on the segmentation process and neural network used. After segmentation process, ANN is performed to distinguish the segmented objects called candidates, as either microcalcifications or nonmicrocalcifications. The accuracy of the ANN is tested by having a set of labelled test images for determination of true positive (TP) and false positive (FP) detection rates. This ANN is using cascade correlation (CC) for pattern classification. It is a self-organizing ANN which runs a supervised learning algorithm. The CC ANN approach shows a promising result to detect microcalcification [16].

Not only image segmentation but also image registration techniques can be used for the breast cancer detection where ANN is performed to enhance the effectiveness of the cancer detection. In Saini and Vijay [17], Grey-Level Cooccurrence Matrix (GLCM) features are extracted and used as input to train artificial neural network based breast cancer detection system. After that, the extracted features of known and unknown mammogram images have been compared using feed-forward backpropagation and Cascade forward backpropagation ANN to distinguish the malignant and benign images. Feed-forward backpropagation network has high accuracy of 87.5% compared to Cascade forward backpropagation network with 67.8% after optimizing the number of neurons and number of layers [17].

In late 90's, the application of ANN in CAD mammography was found to have limitation in terms of data overfitting. Thus, Bayesian belief network (BBN) was compared with ANN classification method to identify the positive mass regions based on a set of computed features in CAD. The same database was used in ANN and a BNN with topologies optimization using a genetic algorithm (GA) to test the performance and robustness of the ANN and BBN. However, the result shows that there is no significant difference between using an ANN and using a BBN in CAD for mass detection if the network is optimized properly [18]. In Alayliogh and Aghdasi [11], wavelet-based image enhancement technique has been used to improve the detection of breast cancer. Input feature vectors containing spatial and spectral image were employed in neural network classifier. Microcalcification detection scheme and wavelet image enhancement have been investigated. Microcalcification detection has been performed by using a multistage algorithm comprising the image segmentation and pattern recognition to classify the microcalcifications whereas biorthogonal spline wavelets have been used in image enhancement to separate the image into frequency bands without affecting the spatial locality. The result shows that spatial and spectral feature are promising ways to detect microcalcification [11].

Besides microcalcification, masses are the most important symptoms which are difficult to be detected and distinguished accurately. A new algorithm based on two ANNs (artificial neural networks) was proposed to detect these masses automatically. ANFIS and multilayer perceptron (MLP) classifier have been used for adjustment and filtration. Suitable methods and parameters should be applied to get high detection precision and low false positive (FP) [10]. The detection process was well adjusted and improved with this proposed algorithm and the final diagnosis result showed that the CAD scheme could simultaneously achieve comparatively high detection precision and low false positive rate, even when the special masses are dealt with [10].

In mammography equipped with CAD system, the major problems developed are inconsistency and low classification accuracy. The accuracy can be improved by introducing a novel intelligent classifier which used texture information as input for the classification of normal and abnormal tissues in mammograms. Dheeba et al. [3] used neutral network as a new artificial intelligent technique for the tissue classification. CAD system based on the optimized wavelet neural network was designed and evaluated using Particle Swarm Optimization approach (PSOWNN). Optimization using heuristic algorithm is done to find appropriate hidden neurons, momentum constant, and learning rate during the training process. Thus, it will improve the classification accuracy in breast cancer detection by reducing the misclassification rate [3]. In Zhang et al. [19], backpropagation neural network (BPNN) is introduced for the classification of benign and malignant. The digitized mammogram used fuzzy detection algorithm to detect the microcalcification and suspicious area. BPNN gives a very promising result with 83.3% for the classification [19].

**Figure 2:**
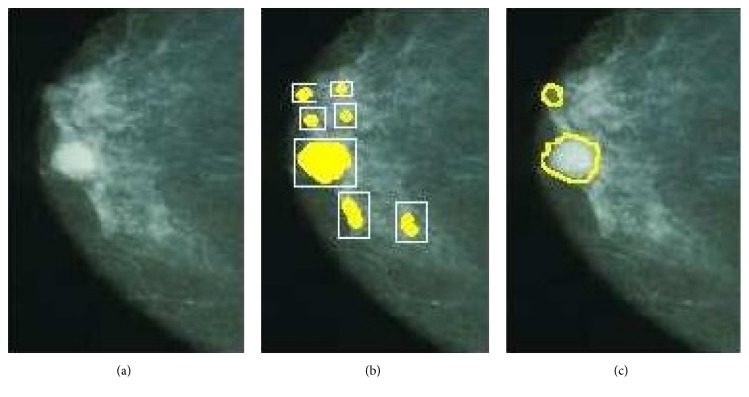
Results (from (a)–(c)): original image, image after first stage of NN processing, and image at second stage of NN processing using Gabor wavelets as input for mammogram image [20].

Any defect in breast image obtained from mammogram is highly advantageous to be detected automatically. In Lashkari [20], Gabor wavelets and ANN are used to classify normal and abnormal tissues which could increase the accuracy and save radiologist's time (Figure 2). Gabor wavelets transforms have a good attribute in image processing and computer vision. The result shows that this combination of neural networks has a good potential with 97% accuracy on unknown cases [20].

### 2.2. Ultrasound

Neural network (NN) also plays its role in ultrasound images in detecting breast cancer. We will first look into the capability of NN in determining and recognizing a region where malignant and benign lesions can be found. Buller [21] was one of the first who used neural network in breast cancer detection for ultrasound images. In his work, he separated the training process for benign and malignant cases by feeding the first system only with images containing benign lesion and the other with images containing only malignant lesion. He also introduces “spider web” topology which are able to produce two vectors that are further used in the classification process. The first vector represent the localized effects in a defined neighborhood and the other represents the global attributes. The technique brings high advantages as the spider web topology is sensitive to small area and hence provides better results in small area by putting more weights on the localized effects. This technique can actually be improved taking into account the extra parameters of texture and shape. As we know, malign and benign lesion has slightly different texture and shape. In Ruggierol et al. [22], the researchers implement the NN using both texture and shape parameters. Transition probability matrix and Run length matrix on the texture parameter have been used to quantify the homogeneity of images while shape parameter shows the irregularity of border of lesion. Besides, three-layer NN consisting of input, output, and hidden neurons was used. They also execute the training process by leaving one out before training and use the left out as tester. From the result, texture implementation achieved a very good result on both solid and liquid lesion.

Classification is an important technique used widely to differentiate cancerous and noncancerous breasts. Denser breast has higher risk in having cancer. Knowing this, Sahiner et al. [23] in their paper describe the importance of texture images in classification of dense breast tissue. They also introduce convolution neural network classifier to replace the backpropagation methods where the images are fed directly into the network. To measure the coarseness of texture, grey-level difference statistics and features are used, whereas spatial grey-level dependence features will be showing the element distribution. The strength of this method is that no image of tumor is fed into the network. Besides, segmentation does not need to be performed beforehand; instead a threshold value will be used. The drawback is the high computational cost which in turn makes the technique probably unsuitable for real-time operation.

As early as 1999, NN classifier has been used with autocorrelation features to classify the breast cancer as benign or malignant. Chen et al. [24] introduced a 25-input node multilayer feed-forward NN which consists of 24-dimensional image feature vector from the ultrasound image, together with a predefined threshold of the input layer to classify the cancer. The introduced system has a relatively high accuracy in classifying malignancies and thus could help inexperienced operators to diagnose complicated ultrasound images. One of the striking advantages of NN is that it could be further optimized by supplying larger set of ultrasound images as the NN is well-trainable.

Self-organizing map (SOM) model is one type of neural networks that can be trained without supervision; it is widely used as a classifier in recent years. It expresses lower dimensional data in a simple geometry compared with the complex high dimensional data such as that in Chen et al. [25]. SOM training is fairly easy as a desired output is not necessary. SOM will automatically choose the closest input vector. The classification of benign and malignant lesion is automated. The only training needed is the data from texture analysis. However, its accuracy is slightly lower than that of the multilayer feed-forward NN introduced by Chen et al. [24] previously. With a high negative predictive value of 98%, this CAD technique of SOM could potentially avoid benign biopsies. In the same year, Chen et al. [26] came out with a HNN diagnostic system. The texture information features are extracted from four ultrasound images. The 2D normalized autocorrelation matrix for the input is modified and 2-phase HNN was used to combine the texture information from all of the ultrasound images. This study proves that the use of all 4 images leads to a more promising result than the case where images are used separately.

Bootstrap is a statistical measure that relies on random sampling with replacement. Combination of bootstrap technique with neural network helps improve the accuracy. Chen et al. [27] implement bootstrap to perform random sampling; the observation was then recomputed. This technique is useful where large amount of training data is not available, as it does not require much training data. However, the reduced amount of data should be compensated by adding image analysis component, in the bootstrap method.

The research done in 2002 used error backpropagation algorithm to train the multilayer perceptron neural network (MLPNN) and resulted in an area index of the receiver operating curve (ROC) of 0.9396 ± 0.0183 [28]. Seven morphological features have been introduced to differentiate benign from malignant breast lesions with the use of MPNN [29]. The morphological features were named as lobulation index (LI), elliptic-normalized skeleton (ENS), elliptic-normalized circumference (ENC), depth to width ratio (D : W), long axis to short axis (L : S), number of substantial protuberances and depressions (NSPD), and the size of lesion. The MPLNN is also tested with different number of hidden neurons but all results lead to a similar performance. Accuracy of the training set and test set is better than SOM and MLP NN with three inputs but on par with the accuracy of NN with 25 autocorrelation features as inputs. Different inputs selected and number of inputs may have an impact on the accuracy of the NN itself regardless of any types of NN techniques.

A year later, a research tested the NN by using only 5 morphological features which are the spiculation, branch pattern, ellipsoid shape, brightness of nodule, and the number of lobulations [30, 31]. Based on these morphological features, the difference of characteristics between the benign and malignant could be seen as follows:

- Spiculation (benign: larger; malignant: smaller)
- Branch pattern (benign: fewer; malignant: more)
- Ellipsoid shape (benign: smaller; malignant: larger)
- Brightness of nodule (benign: larger; malignant: smaller)
- Number of lobulations (benign: fewer; malignant: more)

**Figure 3:**
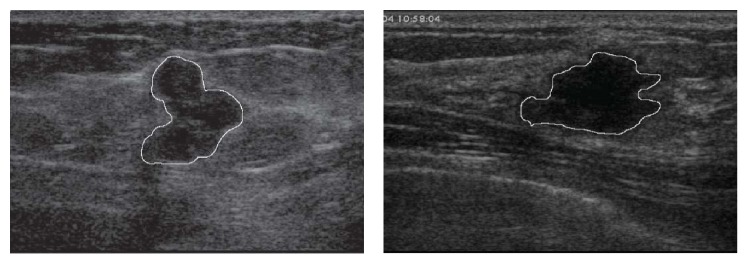
Segmentations of cysts for breast ultrasound image using ANN [32].

Latest research implements the hybrid method to improve the conventional neural network method to detect the malignancies of breast cancer. Combination of the* k*-means cluster algorithm with the backpropagation neural network (BPNN) is proven to provide an impressive performance [32].  Figure 3 shows the result of image segmentation using ANN to extract cysts from an ultrasound breast image [32].

### 2.3. Thermal Imaging

Thermal imaging has been used for early identification of breast tumor and risk prediction since the 60s [33]. Thermogram is a promising cutting edge screening instrument as it can caution ladies of breast malignancy up to 10 years ahead of time [34]. Some studies have utilized several types of ANNs to manipulate and classify IR images, by taking the IR image as an input to the ANN [35]. In 2003, multispectral IR images were classified using Lagrange Constraint Neural Network (LCNN) which provides a better diagnosis for the physician [36]. Wavelet transformation is also useful with ANN for multidimensional features of the IR image, especially when it was found that the temperature of the breast is affected by many pathological factors including the mental state [37]. Asymmetry discrimination between left and right breasts can be done to produce statistical features such as mean temperature and standard deviation that could be utilized as info parameters to a backpropagation ANN [33]. In 2007, thermographic image analysis was done by implementing a special neural network that utilizes some fuzzy logic principles, called Complementary Learning Fuzzy Neural Network (CLFNN). CLFNN takes many factors into account such as family history and temperature difference of the statistical features between contralateral breasts [34]. The system is widely used in several countries at present.

### 2.4. MRI

**Figure 4:**
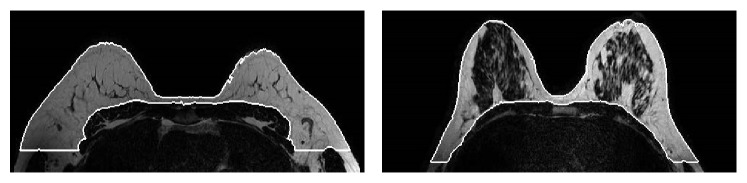
Multistate CNN used to segment small fatty breast and medium dense breast for MRI image [39].

MRI technique has been used widely in medical examinations, especially for cancer investigation for few decades [38]. For the diagnosis to be done properly, breast region should be extracted from other surrounding regions and tissues using image segmentation methods [39].  Figure 4 depicts such case as it was reported in the study [39].

Many neural networks models were utilized to aid MRI for enhancing the detection and the classification of the breast tumors, which can be trained with previous cases that are diagnosed by the clinicians correctly [40], or can manipulate the signal intensity or the mass characteristics (margins, shape, size, and granularity) [41]. In 2012, multistate cellular neural networks (CNN) have been used in MR image segmentation to estimate the density of the breast regions for evaluation of the fat contents [39]. Hassanien et al. [38] introduced a hybrid model consisting of Pulse Couple Neural Network (PCNN) and Support Vector Machines (SVM) to identify breast cancer from MR images. Another hybrid algorithm was presented by ElNawasany et al. in 2014 by combining perceptron with the Scale Invariant Feature Transform (SIFT) for the same purpose [42].

## 3. Discussion

For the last few decades, several computer-aided diagnosis (CAD) techniques have been developed in mammographic examination of breast cancer to assist radiologist in overall image analysis and to highlight suspicious areas that need further attention. It can help radiologist to find a tumor which cannot be spotted using naked eye. As technologies keep growing, many researchers are concerned about the development of intelligent techniques which can be used in mammography to improve the classification accuracy. This artificial intelligence makes use of human skills in a more efficient manner than the conventional mathematical models do. Based on the research outcomes, ANN is proved to be a good classifier in mammography for classification of masses and microcalcifications. Implementation perceptron based three-layer neural network using backpropagation algorithm becomes a pioneer in ANN mammography. Various ANNs developed are based on the concept of increasing the true positive (TP) detection rate and decreasing the false positive (FP) and false negative (FN) detection rate for the optimum result. Implementation of wavelet in ANNs such as Particle Swarm Optimized Wavelet Neural Network (PSOWNN), biorthogonal spline wavelet ANN, second-order grey-level ANN, and Gabor wavelets ANN can improve the sensitivity and specificity which are acquired in masses and microcalcification detection.

For ultrasound applications, in the field of determining breast cancer malignancy, CAD frameworks utilizing ultrasound images are widely used due to their nonradiation properties, low cost, high availability, speedier results, and higher accuracy. An improved version of the breast cancer detection using ultrasound images has been introduced, which works on a three-dimensional ultrasound imaging that can give more in-depth information on the breast lesion compared to the conventional two-dimensional imaging. This three-dimensional imaging joins each of the two-dimensional characteristics. Furthermore, in order to handle the vulnerability nature of the ultrasound images, some methods and methodologies based on ANN have also been introduced. A majority of the research works that utilize ANN have acquired noteworthy results. Hybrid methods, which combine two ANN techniques, have recently been developed for the detection and classification of breast cancer. A two-phase hierarchical NN is also found to be promising rather than using the image analysis separately. It can also be seen that the larger the number of inputs to the ANN, the better the accuracy of the output in identification and classification of breast cancer. However, the number of hidden neurons does not seem to have a big impact on the accuracy of the system. To state which individual ANN is the best is quite subjective depending on the application and various variables to be considered. Most of the ANN techniques for the ultrasound application give good results in terms of accuracy, sensitivity, specificity, positive predictive value, and negative predictive value. Another advantage of using the ANN in determining breast lesion is that the ANN can be trained to produce better accuracy. Besides that, this ANN can be combined together with not only another ANN technique but also other signal processing techniques such as wavelet to produce better results.

For different techniques that have been utilized for the breast cancer imaging, there are different methods of detection and classification according to the input parameters of that technique. For the IR imaging, it has been shown that the detection of the breast cancer depends mainly on the statistical features of the thermal behavior of the tissues (mean, standard deviation, etc.), as well as the asymmetry differentiation of the contralateral breasts. Therefore, image classification methods based on ANN are quite fruitful in thermography.

The MRI imaging is highly recognized as a reliable technique for tumor localization as well as early detection and classification of cancer, as it is generally recommended for soft tissue recognition. Many image segmentation and 3D extraction algorithms are applied in MRI applications, and recently, many ANN classification types have been designed with many fine specifications for MRI breast imaging.

A summary of methods with NN in breast cancer detection has been given in tabulated form in Table 1.

## 4. Conclusion

Neural network plays an important role in detection of carcinogenic conditions in the breast. The technique acts as a stepping stone in the detection of cancer. In this review, we show that NN can be used in many medical applications which we categorized into four main medical applications that are widely used in breast cancer detection. These four medical applications include mammogram, ultrasound, and thermal and MRI imaging. This shows that NN is not restricted by the application.

In all applications, NN's main purposes were automated classification and segmentation. The types of data that need to be classified include calcification and noncalcification, benign and malignant, dense and normal breast, and tumorous and nontumorous. Neural network needs training data. Different types of data are fed into NN for training purposes. In early adaptation of NN, images of breast are being fed directly into the NN. This method will perform well only if very large databases are available. In the case of using such huge data, the concern was the storage, the time of performance, and the data availability. This flaw was realized and being improved by taking the ROI into account, which lowered the amount of dataset requirement tremendously. Researcher were then able to come out with better ideas where they now train the NN with feature vectors. In our findings, the features that can be used as training data include spiculation, branch pattern, shape, brightness of nodule, number of lobulations, margin, size of nodule, granularity, and texture. These features can be extracted manually or using image analysis technique. Introducing of features did improve the performance of NN in terms of size of training data and accuracy.

Different variation of NN can be applied as classifier. Feed-forward backpropagation NN is by far the simplest form of NN, as the name suggest, the input nodes do not have interrelation between each other, and more importantly, the units do not form a repetitive cycle or loops. Feed-forward backpropagation can only pass data from current layer to subsequent layer; hence the data is moving in one fix direction from input to output. Cascade forward NNs are somehow similar to feed-forward NNs; the only difference is that they include connections from not only the input, but also every previous layer to the subsequent layers. Convolution NN is considered as a special type of feed-forward neural network where there are multiple layers of small neuron collections that are able to process the portions of input image.

The trend now is going towards hybrid NN like SOM model. Combination of statistical methods such as bootstrap is being used together with NN too. SOM and bootstrap methods require lesser training data and hence are useful when we do not have many training data. Besides, people utilize SVM with NN in order to achieve a better performance. In conclusion, NN is widely used in medical image applications, creatively combined with other methods in order to achieve better accuracy, sensitivity, and also positive predictive value.

## Figures and Tables

**Table 1:** Summary of methods with NN in breast cancer detection.

Study	Methods	Input	Purpose	Dataset	Classifier	Results
Dheeba et al. [3]	Particle Swarm Optimized Wavelet Neural Network (PSOWNN)	Mammogram	Improve classification accuracy in breast cancer detection and reducing misclassification rate	216 mammograms	PSOWNN	(i) Sensitivity 94.167% (ii) Specificity 92.105% (iii) AUC 0.96853 (iv) Youden's index 0.86272 (v) Misclassification rate 0.063291

Xu et al. [10]	New algorithm based on two ANNs	Mammogram	Classification of masses	30 cases and 60 mammograms (containing 78 masses)	ANFIS and MLP	(i) True positive (TP) rate 93.6% (73/78), (ii) Number of the FPs per image 0.63 (38/60).

Alayliogh and Aghdasi [11]	ANN and biorthogonal spline wavelet	Mammogram	Classification of microcalcification cluster (MCC) and image enhancement	40 digitized mammogram	ANN	(i) Sensitivity 93%, (ii) FP rate (MCC/image) 0.82

Dhawan et al. [13]	(i) ANN (ii) second-order gray-level statistics	Mammogram	Classification of significant and benign microcalcifications	5 image structure features	(i) Three-layer perceptron based ANN	The entropy feature has significant discriminating power for classification

Chitre et al. [15]	ANN	Mammogram	Classification of microcalcification into benign and malignant	(i) 40, 60, and 80 training cases (ii) 151, 131, and 111 test cases	ANN	Neural network is a robust classifier of a combination of image structure and binary features into benign and malignant

Kevin et al. [16]	ANN	Mammogram	Classification of microcalcifications and nonmicrocalcifications	24 mammograms with each containing at least one cluster of microcalcifications	Cascade correlation ANN (CC ANN)	(i) TP detection rate for individual microcalcifications is 73% and 92% for nonmicrocalcifications

Zheng et al. [18]	ANN and BBN	Mammogram	Compare performances of ANN and BBN	3 independent image databases and 38 features	ANN and BBN	Performance level (*A*_*z*_ value) (i) ANN *A*_*z*_ value 0.847 ± 0.014(ii) BBN *A*_*z*_ value 0.845 ± 0.011 (iii) Hybrid classifier (ANN and BBN) *A*_*z*_ value increased to 0.859 ± 0.01

Zhang et al. [19]	Digitize module, detection module, feature extraction module, neural network module, and classification module	Mammogram	Classification of microcalcification clusters/suspicious areas	Fuzzy detection algorithm (i) 30 digital images (15 contain benign cases and 15 contain malignant cases)	Backpropagation neural network (BPNN)	(i) Fuzzy detection rate (benign 84.10% and 80.30%) (ii) Classification rates (feature vector, *n* = 10 is 83.8%), (feature vector *n* = 14 is 72.2%)

Lashkari [20]	ANN and Gabor wavelets	Mammogram	Classification of breast tissues to normal and abnormal classes automatically	(i) Images of 50 normal and 50 abnormal breast tissues (ii) 65 cases for training set and 35 cases for testing set	ANN and Gabor wavelets	(i) Classification rate (testing performance 96.3% and training performance 97.5%)

Saini and Vijay [17]	Image registration technique and ANN	Mammogram	Classification of benign and malignant	42 mammogram images (30 benign and 12 malignant images)	Feed-forward backpropagation and Cascade forward backpropagation artificial neural network	Percentage accuracy (feed-forward backpropagation network is 87.5% and Cascade forward backpropagation network is 67.8%)

Buller et al. [21]	Spider web topology with NN	Ultrasound	Classify and separate benign and malignant lesion	25 sonograms	(i) NN classifier	(i) 69% accuracy in malignant (ii) 66% accuracy in benign (iii) 66% accuracy in no lesions

Ruggierol et al. [22]	Texture and shape parameter feeds into NN	Ultrasound	Automated recognition of malignant lesion	(i) 41 carcinomas (ii) 41 fibroadenomas (iii) 41 cysts	(i) NN classifier	(i) 95% accuracy in solid lesions (ii) 92.7% accuracy in liquid lesions

Sahiner et al. [23]	Convolutional NN with spatial and texture image	Mammogram	Classification of mass and normal breast	168 mammograms	(i) Convolution NN classifier	(i) Average true positive fraction of 90% at false positive fraction of 31%

Chen et al. [24]	Multilayer feed-forward neural network (MFNN)	Ultrasound	Classify benign and malignant lesion	140 pathological proved tumors (52 malignant, 88 benign)	MFNN	(i) 95% accuracy, 98% sensitivity (ii) 93% specificity (iii) 89% positive predictive value (iv) 99% negative predictive value

Chen et al. [25]	Self-organizing map (SOM)	Ultrasound	Classification of benign and malignant lesions	243 tumors (82 malignant, 161 benign)	SOM	(i) Accuracy of 85.6, sensitivity 97.6% (ii) Specificity 79.5% (iii) Positive predictive value 70.8% (iv) Negative predictive value 98.5%

Chen et al. [27]	Bootstrap with NN	Ultrasound	classification of tumor	263 sonographic image solid breast nodules	NN	(i) Accuracy 87.07%, sensitivity 98.35% (ii) Specificity 79.10% (iii) Positive predictive value 81.46% (iv) Negative predictive value 94.64%

Chen et al. [26]	2-phase Hierarchical Neural Network (HNN)	Ultrasound	Differentiate between benign and malignant tumors	1020 images (4 different rectangular regions from the 2 orthogonal planes of each tumor)	HNN	4 image analyses of each tumor appear to give more promising result than if they are used separately

Chen et al. [28]	Wavelet transform and neural network	Ultrasound	Differential diagnosis of breast tumors on sonograms	242 cases (161 benign, 82 malignant)	Multilayer perceptron neural network (MLPNN)	(i) Receiver operating characteristic (ROC) area index is 0.9396 ± 0.0183 (ii) 98.77% sensitivity, 81.37% specificity (iii) 72.73% positive predictive value (iv) 99.24% negative predictive value

Chen et al. [29]	Multilayer feed-forward neural network (MFNN)	Ultrasound	Differentiate benign from malignant breast lesions	1st set: 160 lesions 2nd set: 111 lesions	MFNN	(i) 98.2% training accuracy (ii) 95.5% testing accuracy

Joo et al. [30]	Artificial neural network (ANN)	Ultrasound	Determining whether a breast nodule is benign or malignant	584 histologically confirmed cases (300 benign, 284 malignant)	ANN	(i) 100% training accuracy (ii) 91.4% testing set (iii) 92.3% sensitivity, 90.7% specificity

Joo et al. [31]	Digital image processing and artificial neural network	Ultrasound	Determine breast nodule malignancy	584 histologically confirmed cases (300 benign, 284 malignant)	ANN	(i) 91.4% accuracy, 92.3% sensitivity (ii) 90.7% specificity

Zheng et al. [32]	Hybrid method (unsupervised *k*-means cluster, supervised backpropagation neural network (BPNN))	Ultrasound	Classification of breast tumors as benign or malignant	125 benign tumors, 110 malignant tumors	Combination of *k*-means with BPNN	(i) Recognition rate (94.5% for benign, 93.6% for malignant) (ii) 94% accuracy, 94.5% sensitivity (iii) 93.6% specificity

Fok et al. [35]	ANN with 3D finite element analysis	IR	Tumor prediction	200 patients	ANN	Good detection, poor sensitivity

Szu et al. [36]	Unsupervised classification using Lagrange Constraint Neural Network (LCNN)	Mid and long IR images	Early detection of breast cancer	One patient with DCIS	LCNN	Better sensitivity

Jakubowska et al. [37]	ANN with wavelet transform	IR	Discrimination of healthy and pathological cases	30 healthy	ANN	Accuracy (%) (frontal/side) Raw: 90/93, PCA: 90/93 LDA: 93/97, NDA: 93/93
				10 with recognized tumors		Accuracy (%) (frontal/side) Raw: 80/90, PCA: 80/90 LDA: 90/90, NDA: 80/100

Koay et al. [33]	Backpropagation NN	IR	Early detection of breast cancer	19 patients	Levenberg-Marquardt (LM) and Resilient Backpropagation (RP)	Accuracy (%) (RP/LM) Whole: 95/95 Quadrants: 95/100

Tan et al. [34]	Fuzzy adaptive learning control network fuzzy neural network	IR	Early detection of breast cancer and tumor classification	28 healthy, 43 benign tumors, 7 cancer patients	FALCON-AART	Cancer detection (%) (TH/TDF) Predicted: 95, sensitivity: 100, specificity: 60 Breast tumor detection (%) (TH/TDF) Predicted: 84/71, sensitivity: 33/76, specificity: 91/62 Breast tumor classification (%) (TH/TDF) Predicted: 88/84, sensitivity: 33/33, specificity: 95.5/91

Cardillo et al. [40]	NN for automatic analysis of image statistics	MRI	Early detection and classification	150 exams subdivided into 6 groups by contrast	NN	Better in specificity

Tzacheva et al. [41]	Evaluation of signal intensity and mass properties by NN	MRI	Automatic diagnosis of tumors	14 patients	Feed-forward BPNN	90%–100% sensitivity, 91%–100% specificity, and 91%–100% accuracy

Ertas et al. [39]	Extraction of breast regions by conventional and multistate CNNs	MRI	Breast density evaluation and abnormality localization	23 women	CNN	Average precision 99.3 ± 1.8% True positive volume fraction 99.5 ± 1.3% False positive volume fraction 0.1 ± 0.2%

Hassanien et al. [38]	Image classification using PCNN and SVM and using wavelet and fuzzy sets for enhancement	MRI	Breast cancer detection	70 normal cases, 50 benign and malign cases	Hybrid scheme of PCNN and SVM	Accuracy SVM: 98% Rough sets: 92%

ElNawasany et al. [42]	Classifying MR images by hybrid perceptron NN	MRI	Early detection of breast cancer	138 abnormal and 143 normal	Perceptron with SIFT	Accuracy 86.74%
